# Supplementary material for: The Molecular Bases of the Dual Regulation of Bacterial Iron Sulfur Cluster Biogenesis by CyaY and IscX
Source: Front Mol Biosci. 2018 Feb 2;4:97. doi: 10.3389/fmolb.2017.00097 (PMC5801593; doi:10.3389/fmolb.2017.00097)
Supplement: Supplementary file 1 [file Image1.PDF]

Supplementary Materials

Mass spectrometry under non-denaturing conditions

Proteins were exchanged into 250 mM ammonium acetate, pH 8.0 using Zeba spin (~7 kDa MWCO, Thermo Scientific) and illustra Micro spin G-25 (~3 kDa MWCO, GE Healthcare) for IscS or IscX, respectively. The volume of the eluent was increased to 1 ml for IscS. Solutions of IscS (3 μM) were mixed with increasing concentrations of IscX (0–16 IscX-IscS molar ratios). Samples were incubated at room temperature for 5 min before being loaded in a 500 μl gas-tight syringe (Hamilton) and infused directly, via a syringe pump (0.3 ml/hr), in a Bruker micrOTOF-QIII mass spectrometer (Bruker Daltonics) operating in the positive ion mode. The ESI-TOF was calibrated online using ESI-L Low Concentration Tuning Mix (Agilent Technologies). MS data were acquired over the m/z range 4,000–8,000 continuously for 10 min, with acquisition controlled using Bruker oTOF Control software, dry gas flow 3 L/min, 190 °C, nebulizer gas pressure 0.8 Bar, capillary voltage of 3,200 V, offset of 500 V, ion energy 6 eV, collision radio frequency of 3000 Vpp, and collision cell energy of 10 eV. Optimization of experimental conditions for the transmission of dimeric IscS or IscX-IscS complexes was achieved by increasing the equivalent of the cone-voltage (in-source collision induced dissociation (isCID)) to 135 eV(1). For LC-MS, an aliquot of IscS or IscX was diluted with an aqueous mixture of 2% (v/v) acetonitrile, 0.1% (v/v) formic acid, and loaded onto a Proswift RP-1S column (4.6 x 50 mm, Thermo Scientific) attached to an Ultimate 3000 uHPLC system (Dionex, Leeds, UK). Bound proteins were eluted (0.2 ml/min) using a linear gradient (15 min) from 2% to 100% (v/v) acetonitrile, 0.1% (v/v) formic acid, and infused directly into the source of the mass spectrometer, as previously described(2). Processing and analysis of MS experimental data were carried out using Compass DataAnalysis v4.1 (Bruker Daltonik). Before processing, data sets spanning the 4000 –8000 m/z region were re-calibrated off line with the cesium salt of perfluoroheptanoic acid(3, 4) and a 0.49 m/z Gaussian smoothing algorithm was applied. Neutral mass spectra were generated using the ESI Compass v1.3 Maximum Entropy deconvolution algorithm over the mass range of 90,000 Da to 140,000 Da or over more specific ranges covering masses of interest. Exact masses are reported from peak centroids representing the isotope average neutral mass. Predicted masses are given as the isotope average of the neutral protein or sum of the protein complex. Titration data were fitted according to the Scheme below:

$$[X] + [S]_2 \leftrightarrow [X]{:}[S]_2 \qquad K_{d1}$$
$$[X] + [X]{:}[S]_2 \leftrightarrow [X]_2{:}[S]_2 \qquad K_{d2}$$
$$[X] + [X]_2{:}[S]_2 \leftrightarrow [X]_3{:}[S]_2 \qquad K_{d3}$$
$$[X] + [S]_2{:}[X]_3 \leftrightarrow [X]_4{:}[S]_2 \qquad K_{d4}$$

where S and X represent IscS and IscX, respectively. The data were fitted using the program DynaFit (BioKin, CA, USA) which, by solving simultaneous non-linear algebraic equations, can determine the composition of complex mixtures at equilibrium.

Fragment identification by Mass spectrometry

Following the cross-linking reaction, protein samples were loaded on a 10% SDS gel to separate isolated IscS from the covalent complexes. Each gel band underwent trypsin in gel digestion, followed by MALDI/MS analyses. A comparison between the MALDI/MS spectra acquired for the protein and for the protein complexes allowed identification of the putative cross-linked peptides, whose identity was confirmed by high resolution LC/MS and LC/MS/MS. Mass spectra were acquired in positive reflector and linear mode on a MALDI micro MX (Waters, Milford, USA). External calibration using peptides derived by tryptic digestion of lactoglobulin (Sigma-Aldrich, Milano, Italy) was performed. Processing of the MS spectra was performed by MassLynks data processor. High resolution LC/MS and LC/MS/MS analyses were carried out using a LTQ Orbitrap XL ESI-mass spectrometer (Thermo Fisher Scientific) equipped with a nano-ESI source, coupled with a nano-Aquity capillary UPLC (Waters). Peptides separation was performed on a capillary BEH C18 column (0.075 mm×100 mm, 1.7 µm, Waters) using aqueous 0.1% formic acid (A) and CH_3CN containing 0.1% formic acid (B) as mobile phases. Peptides were eluted by means of a linear gradient from 10% to 40% of B in 45 min and a 300 nl•min^{-1} flow rate. Mass spectra were acquired in a *m*/*z* range from 400 to 1800 and MS/MS spectra from 25–2000. Calibration was performed using NaI clusters as external standard and [Glu]-Fibrinopeptide B human (Sigma-Aldrich, Milano, Italy) as lock mass standard.

SAXS data analysis

The forward scattering *I(0)* and the radius of gyration R_g were evaluated using the Guinier approximation(5) which assumes that at very small angles ($s < 1.3/R_g$) the

intensity is represented as $I(s) = I(0) \exp(-1/3(R_g s)^2)$. These parameters were also computed from the entire scattering pattern using the program GNOM(6), which provides the distance distribution functions $p(r)$ and the maximum particle dimensions D_{max}. The molecular mass (MM) of the solute was estimated by normalization of $I(0)$ against reference solutions of bovine serum albumin. The excluded volume of hydrated particle was computed as reported by Porod(7).

Molecular modelling for the IscX-IscS complex with a 1:1 molar ratio was done using the theoretical models of the IscS dimer (PDB code: 3LVL) and the monomeric IscX (PDB code: 2BZT) with the program SASREFMX(8) taking into account the possible presence of the full subunit complex (the IscS dimer with four IscX molecules) and its dissociated part (IscS dimer with only two IscX molecules). The fit quality to the experimental data $I_{\exp}(s)$ is assessed by minimizing the discrepancy:

$$\chi^2 = \frac{1}{N-1}\sum_j \left[\frac{I(s_j) - cI_{calc}(s_j)}{\sigma(s_j)}\right]^2 \qquad (1)$$

where N is the number of experimental points, c is a scaling factor, $I_{calc}(s_j)$ and $\sigma(s_j)$ are the calculated intensity and the experimental error at the momentum transfer s_j, respectively. The scattering amplitudes from the high resolution models were calculated with the CRYSOL software(9). A P2 symmetry was applied during the modelling. The interaction sites between IscS and IscX determined here and by previous structural and mutagenesis studies were used as structural restraints(10, 11). The partial dissociation of the IscX-IscS complex was allowed to account for two binding sites of IscX with different K_d values. The program OLIGOMER(12) was used to account for complex formation and fit the data obtained at different molar ratios. These solutions could contain the possible components including individual IscX monomers, IscS dimers as well as 1:1 complex (partially dissociated complex of IcsS dimer with two IscX monomers) and a full 2:1 complex of IscS dimer with four IscX monomers. Given the scattering curves of the components, OLIGOMER finds their volume fractions by solving a system of linear equations to minimize the discrepancy (1) between the experimental data and the calculated curve from the mixture. The models of the complexes were taken from ten independent runs of SASREFMX.

Table 1 – Summary of the predicted and observed masses of the different species.

Species	Predicted Mass (Da)	Average Observed Mass (Da)	ΔMass (Da)
**Monomeric Species**			
Apo-IscS	45,289	45,289[a]	0
Holo-IscS	45,519	45,519[b,c]	+1
IscX	7,859	7,935[a,b]	+76[d]
**Dimeric Species** [b]			
Holo-$(IscS)_2$	91,037	91,035	-2
**Complexes** [b]			
$(IscS)_2(IscX)$	98,972[e]	98,973	+1
$(IscS)_2(IscX)_2$	106,907[e]	106,910	+3
$(IscS)_2(IscX)_3$	114,842[e]	114,858	+16[f]
$(IscS)_2(IscX)_4$	122,777[e]	122,793	+16[f]

a. Determined by LC-MS.
b. Determined by native ESI-MS.
c. The mass of pyridoxal phosphate in the lysine-aldimine form is 230 Da.
d. Most likely due to a *β*-mercaptoethanol adduct.
e. Re-calculated using the observed mass for IscX (7935 Da).
f. This may be an oxygen adduct.

Table S2 – Results of the MALDI/MS analyses of peptides produced by in gel trypsin digestion of the IscS-IscX complexes.

**Peptide**	*Experimental M.W. (MALDI)*	*Experimental M.W. (HR LC-MS)*	*Theoretical M.W*
	IscS		
**3-18**	1819.7	1819.889[a]	1819.936
**23-39**	1920.7	1920.751	1920.799
**43-55**	1490.7	1490.582	1490.715
**56-67**	1267.5	1267.628	1267.652
**68-84**	1793.8	1793.836	1793.905
**106-112**	776.4		776.385
**117-128**	1408.6	1408.671	1408.699
**129-135**		771.432	771.449
**136-142**	818.4		818.4
**174-187**	1486.6	1486.713	1486.778
**188-196**	1025.6	1025.583	1025.612
**197-206**	1119.4	1119.501	1119.538
**226-237**	1320.5	1320.565	1320.599
**241-257**	1813.8	1813.866	1813.914
**261-269**	1124.4	1124.410	1124.448
**277-282**	729.4		729.417
**283-318**		4004.853	4004.972
**319-340**	2211.7	2211.908	2212.068
**341-354**	1494.6	1494.713	1494.779
**360-374**	1856.6	1856.861	1856.904
**382-391**	1280.5	1280.592	1280.611
**392-404**	1504.4	1504.654	1504.706
	IscX		
**10-25**	1807.8	1807.814	1807.851
**29-52**	2898.2	2898.123	2898.175
**53-66**	1625.8	1625.841	1625.892
	Cross-linked peptides		
**(85-101)-(*1-9*)**	3120.6	3120.546	3120.620
**(96-105)-(*1-9*)**	2423.2	2423.204	2423.285

[a]The decimal digits are within the precision of this technique.

Table S3 – Overall parameters calculated from SAXS. R_g is the radius of gyration; D_{max} the maximum size of the particle; V_p the excluded volume of the hydrated particle; MM_{exp} the experimental molecular mass of the solute and χ_{rb} the values for the fit curves from rigid body models of the complexes using SASREFMX.

**Sample (molar ratio)**	**c/mg/ml**	**R_g, nm**	**D_{max}, nm**	**V_p, nm ^{3}**	**MM_{exp}, kDa**	χ^2_{rb}
IscX-IscS (1:1)	10	3.04±0.04	10.6±0.5	122±10	84±5	3.35
IscX-IscS (1:1)	5	3.08±0.04	11.0±0.5	132±10	92±5	1.72
IscX-IscS (1:1)	3	3.09±0.04	11.1±0.5	135±10	93±5	1.32
IscX-IscS (2:1)	3	3.08±0.04	11.0±0.5	129±10	87±5	1.22
IscX-IscS (20:1)	0.5	3.01±0.03	10.3±0.5	56±5	40±10	n/a
IscX-IscS (40:1)	0.5	2.75±0.03	10.2±0.5	31±4	25±10	n/a
IscS	3	3.09±0.04	10.8±0.5	136±10	89±5	n/a

Table S4. SAXS data fitting results for the estimate of volume fractions in the mixtures of IscX-IscS at different molar ratios and concentrations obtained by OLIGOMER. The values in parentheses correspond to the mixture analysis with only one type (2:1) of IscX-IscS complexes.

Sample (molar ratio)	Concentration (mg/ml)	Free IscX, %	Free IscS, %	Complex (1:1), %	Complex (1:2), %	χ^2
IscX-IscS (1:1)	10	4 (10)	34 (15)	10 (0)	52 (75)	2.85 (3.24)
	5	8 (11)	24 (8)	33 (0)	35 (81)	1.44 (1.63)
	3	6 (6)	30 (50)	23 (0)	41 (44)	1.19 (1.23)
IscX-IscS (2:1)	3	15 (14)	10 (8)	2 (0)	73 (78)	1.11 (1.12)
IscX-IscS (20:1)	0.5	66 (65)	8 (10)	1 (0)	25 (25)	1.05 (1.06)
IscX-IscS (40:1)	0.5	84 (83)	0 (2)	1 (0)	15 (15)	1.04 (1.05)

Supplementary Figures

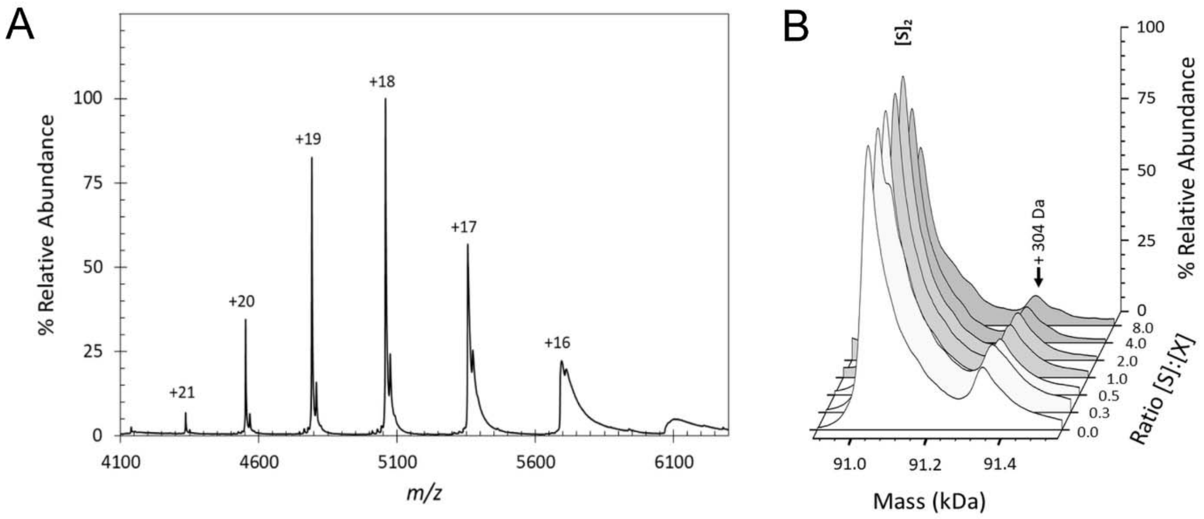

Figure S1. ESI-MS of IscS. (A) m/z spectrum of IscS, revealing charge states due to the IscS dimer species. (B) Deconvoluted mass spectra of IscS measured with increasing concentrations of IscX; the plot shows data for IscX:IscS molar ratios from 0–8. A low intensity peak was observed at +304 Da, due to an unknown adduct of the IscS dimer, as discussed in the main paper. IscS (3 µM) was in 250 mM ammonium acetate, pH 8.

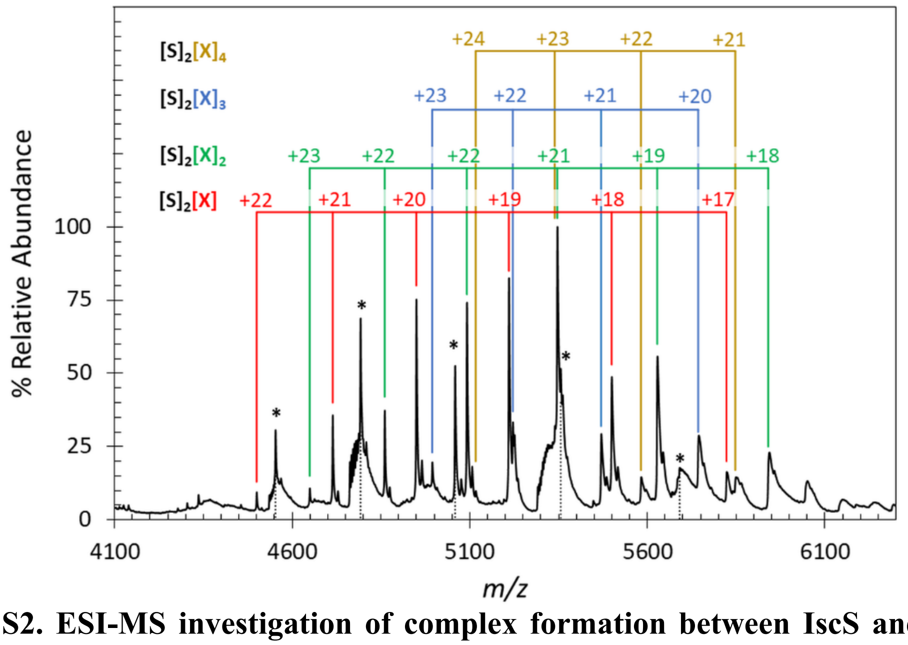

Figure S2. ESI-MS investigation of complex formation between IscS and IscX. Spectrum of a solution of IscX and IscS (8:1 ratio) measured under non-denaturing conditions. Charge states due to four different complexes, (IscX)(IscS)$_2$ to (IscX)$_4$(IscS)$_2$, as well as the uncomplexed IscS dimer, were detected. Deconvoluted spectra are shown in Figure 3 of the main paper. Asterisks mark charge states due to (IscS)$_2$.

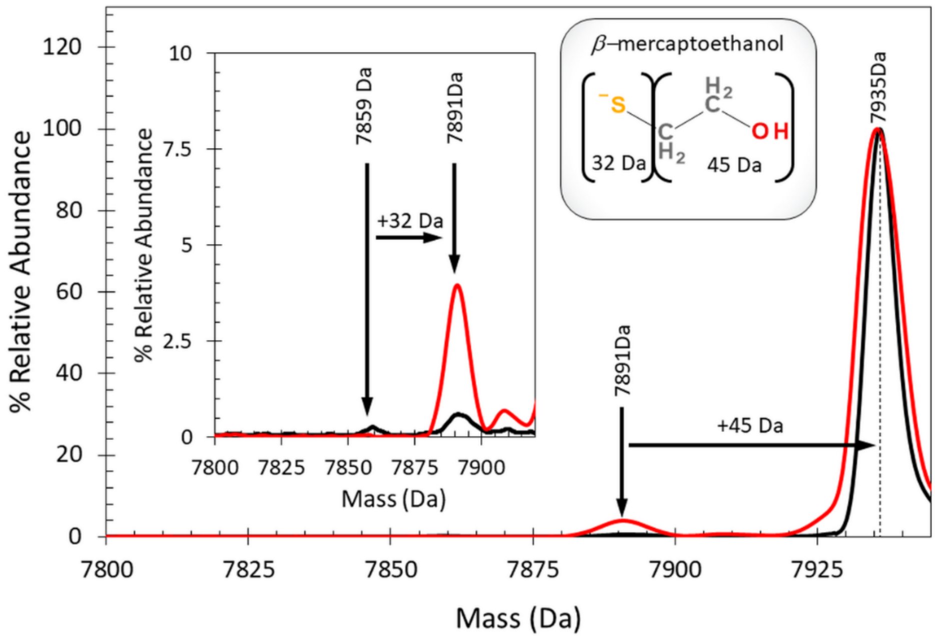

Figure S3. ESI-MS analysis of IscX. Both LC-MS and MS under non-denaturing conditions revealed a mass of IscX of 7935 Da, i.e. 76 Da higher than that predicted by sequence (black spectrum shows the deconvoluted LC-MS). A low intensity peak (<1%) was observed at 7859 Da (the predicted mass), indicating that the main protein peak represents an adduct of the protein. Since the protein was in a buffer containing 20 mM β-mercaptoethanol, a hetero-disulfide between IscX and β-mercaptoethanol is a good candidate for the adduct (predicted mass of 7859 + 78 – 2 = 7935 Da). To test this further, LC-MS was performed with CID (between 35 and 70 eV), which resulted in the spectrum shown in red. Here, a peak at +32 Da relative to the predicted mass and -45 Da relative to the main adduct peak was observed at significantly increased intensity. This is entirely consistent with the presence of a β-mercaptoethanol adduct, with partial fragmentation occurring during collision, causing loss of an ethanol moiety to generate a sulfur bound form of the protein at +32 Da.

A **B**

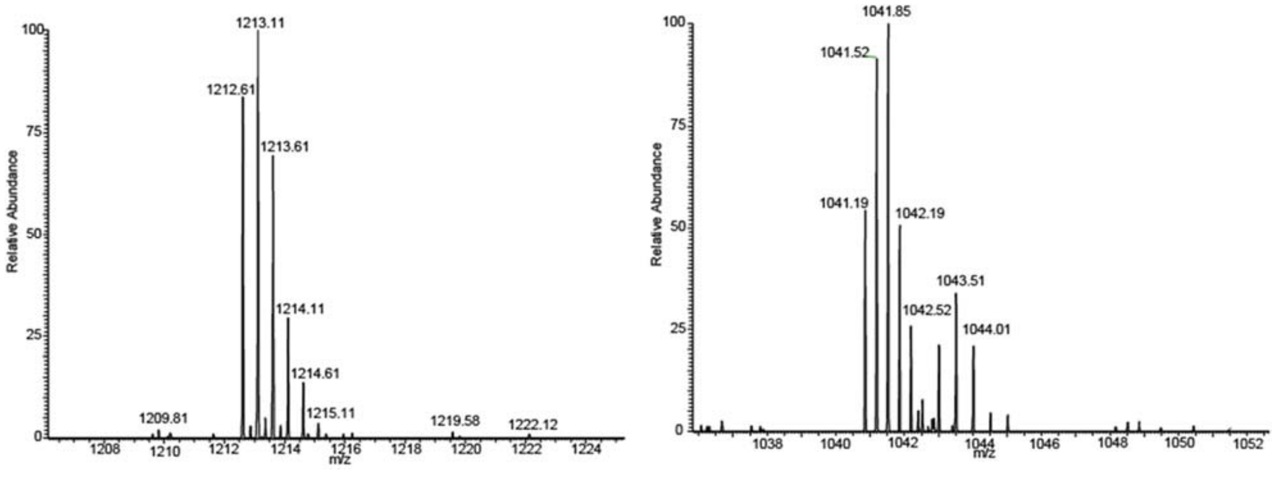

Figure S4 – MS spectra of the cross-linked peptides. The fragment (96-105)-(1-9) ($[M+2H]^{2+}$ is shown in panel A), (85-101)-(1-9) ($[M+3H]^{3+}$ in panel B).

MS/MS of 1041.6

Fragment	m/z $[M+2H]^{2+}$
b_{16}	1479.8
y_{16}	1533,4
y_{15}	1497,9
y'_{8}	1496,4
b'_{8}	1466,2
b_{15}	1435,7
$y_{15}y'_{8}$	1432,4
b'_{7}	1422,7
y'_{6}	1411,3
y_{13}	1405,4
$b_{16}b'_{8}$	1401,6
b_{14}	1379,3
$b_{15}b'_{8}/b_{16}b'_{7}$	1358,3
$y_{15}y'_{6}$	1347,3
$y_{13}y'_{8}$	1339,8
y_{12}	1331,8
$y_{12}y'_{8}$	1266,3
$y_{13}y'_{6}$	1254,7
$y_{11}y'_{8}$	1184,8
$y_{12}y'_{6}$	1181,2
$y_{11}y'_{6}$	1099,7

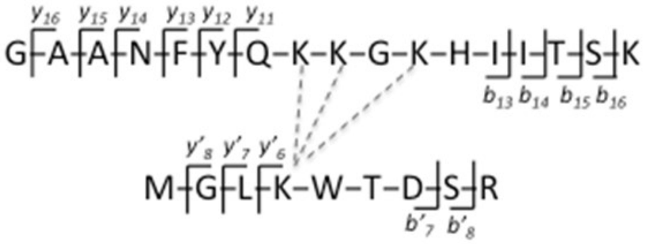

Figure S5 – MS/MS data obtained from the fragmentation of the $[M+3H]^{3+}$ ion at *m/z* 1041.6 and assigned to the reported cross-linked peptide. The observed $[M+2H]^{2+}$ fragments confirmed the identity of the analysed species.

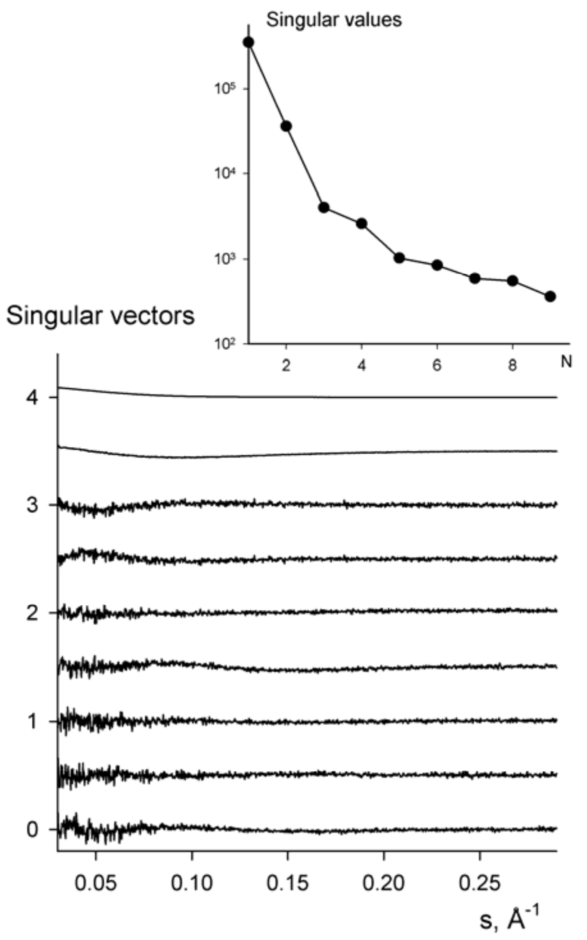

Figure S6 – Singular value decomposition (SVD) of SAXS data for IscX-IscS recorded at different concentrations (10, 5 and 3 mg/ml) and different stoichiometries (1:1, 2:1, 20:1 and 40:1). The insert shows singular values sorted in the descending order, the main graph displays the corresponding singular vectors. Only four non-randomly oscillating singular vectors (the first four vectors from the top of the main graph) are found. It suggests that the system can be described by four independent components contributing to the scattering data, thus validating our choice of free IscS, free IscX and their 1:1 and 2:1 complexes for SAXS data modelling.

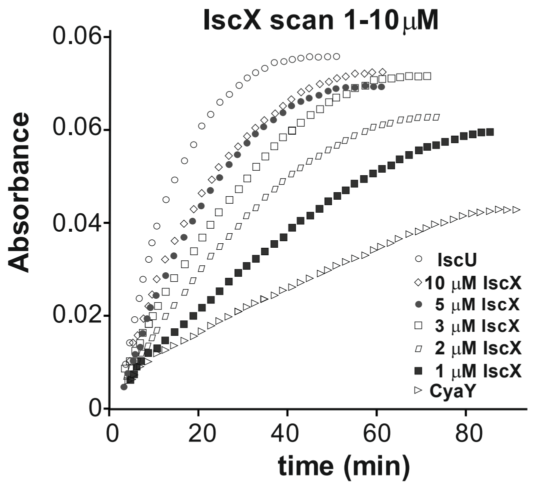

Figure S7 – Competition between IscX and CyaY. A) The same experiment shown in Figure 5A carried out using 25 μM Fe^{2+} in the co-presence of CyaY (5 μM) but increasing the concentrations of IscX only from 0 to 10 μM. As a control, the experiment was carried out with no CyaY and no IscX (open circles). Under these conditions, only the first binding site of IscX on IscS is populated.

References

1. Laganowsky A, Reading E, Hopper JT, Robinson CV. Mass spectrometry of intact membrane protein complexes. Nature protocols. 2013 Apr;8(4):639-51. PubMed PMID: 23471109. Pubmed Central PMCID: 4058633.
2. Crack JC, Thomson AJ, Le Brun NE. Mass spectrometric identification of intermediates in the O2-driven [4Fe-4S] to [2Fe-2S] cluster conversion in FNR. Proceedings of the National Academy of Sciences of the United States of America. 2017 Apr 18;114(16):E3215-E23. PubMed PMID: 28373574. Pubmed Central PMCID: 5402453.
3. Konig S, Fales HM. Calibration of mass ranges up to m/z 10,000 in electrospray mass spectrometers. J Am Soc Mass Spectr. 1999 Mar;10(3):273-6. PubMed PMID: WOS:000078807100012. English.
4. Jackson VA, Mehmood S, Chavent M, Roversi P, Carrasquero M, Del Toro D, et al. Super-complexes of adhesion GPCRs and neural guidance receptors. Nature communications. 2016 Apr 19;7:11184. PubMed PMID: 27091502. Pubmed Central PMCID: 4838878.
5. Guinier A. La diffraction des rayons X aux tr`es petits angles: application `a l'étude de phénom`enes ultramicroscopiques [Th`ese]. Paris,: Univ. de Paris.; 1939.
6. Svergun DI. Determination of the regularization parameter in indirect-transform methods using perceptual criteria. J Appl Crystallogr. 1992;25:495-503.
7. Porod G. General theory. In: Glatter O, Kratky O, editors. Small-angle X-ray scattering. London: Academic Press; 1982. p. 17-51.
8. Petoukhov MV, Franke D, Shkumatov AV, Tria G, Kikhney AG, Gajda M, et al. New developments in the ATSAS program package for small-angle scattering data analysis. Journal of Applied Crystallography. 2012;45(2):342-50.
9. Svergun DI, Barberato C, Koch MHJ. CRYSOL - a program to evaluate X-ray solution scattering of biological macromolecules from atomic coordinates. J Appl Crystallogr. 1995;28:768-73.
10. Kim JH, Bothe JR, Frederick RO, Holder JC, Markley JL. Role of IscX in iron-sulfur cluster biogenesis in Escherichia coli. Journal of the American Chemical Society. 2014 Jun 04;136(22):7933-42. PubMed PMID: 24810328. Pubmed Central PMCID: 4063190.
11. Pastore C, Adinolfi S, Huynen MA, Rybin V, Martin S, Mayer M, et al. YfhJ, a molecular adaptor in iron-sulfur cluster formation or a frataxin-like protein? Structure. 2006 May;14(5):857-67. PubMed PMID: 16698547.
12. Konarev PV, Volkov VV, Sokolova AV, Koch MHJ, Svergun DI. PRIMUS - a Windows-PC based system for small-angle scattering data analysis. J Appl Crystallogr. 2003;36:1277-82.
